# 

**Published:** 1866

**Authors:** 


### Books and Pamphlets Received.

A Practical Treatise on Urinary and Renal Diseases, including Urinary Deposits, illustrated by numerons cases and engravings. By William Roberts, M. D. Philadelphia: Henry C. Lea, 1866.

Bailliere's American and European Literary Bulletin. Bailliere Brothers, Booksellers and importers, 520 Broadway, New York.

Hygienic Experience in New Orleans during the War, illustrating the importance of efficient sanitary regulations.

Catalogue of the Officers and Students of the University of Michigan, with a statement of the courses of instruction in the various departments—1866.

An nual Report of the Surgeon General United States Army, 1865.

An Essay on the Law of Muscular Action. By Louis Mackall, M. D. Second edition, corrected and revised, 1865.

An Essay on the Life in Nature, by Louis Mackall, M. D., author of "Notes on Carpenter's Human Physiology," etc.

Extract from an unpublished Essay on Physical Force. By Louis Mackall, M. D.

A Communication from the City Physician on Asiatic Cholera. Is it a contagious disease? Boston, 1866.

A Sermon on occasion of the death of Thomas W. Blatchford, M. D., delivered in the Second Street Presbyterian Church, Troy. By D. Kennedy, D. D.

Memorial on the late Thomas W. Blatchford, M. D., read at a meeting of the Governors of the Marshall Infirmary, Troy, N. Y., January 29, 1866. By James Thorn, M. D.

